# Buffalo Medical and Surgical Association

**Published:** 1880-11

**Authors:** 


### Society Reports.

---

### BUFFALO MEDICAL AND SURGICAL ASSOCIATION.

*Stated Meeting, October 5, 1880.*

THE PRESIDENT, DR. LUCIEN HOWE, IN THE CHAIR.

MEMBERS present: Drs. Macniel, Cronyn, Rochester, Wyckoff, Moody, Phelps, Pitt, Brecht, O'Brien, Granger, Kilbourne, Fowler, Keene, Peterson and Burt, and others by invitation.

After transacting certain routine business, the paper for the evening was presented by Prof. Kellicott, of the State Normal School, on "Easy Methods for Detecting Blood-stains." [This is given as the second article in the present issue of the JOURNAL.]

In the discussion which followed, Dr. Rochester acknowledged the success which had crowned the efforts of microscopists and chemists in this department, but thought there was opportunity for much further discovery. It was a great advance to be able in a short time, and by comparatively simple means, to determine the character of a suspected stain, as to whether or not it was blood. Infinitely more desirable, however, was it to so improve the methods of investigation as to say, with certainly, what kind of blood it was.

An instance was cited, where a man on trial for murder, asserted that the stains on his clothes were caused by the blood of a chicken recently killed. No microscope or chemical reaction, could establish the fact of this being human blood, though of course such a differential diagnosis was in the highest degree desirable.

Dr. Cronyn also expressed his gratification at the satisfactory character of the demonstrations accompanying the paper, as he considered the subject one of decided practical importance.

Voluntary communications being next in order, the association first listened to the report of a case from Dr. Fowler. [His paper, like the first, appears in another part of this JOURNAL as a "Clinical Report."] Some discussion ensued concerning the exciting causes of tetanus, after which Dr. Howe called Dr. O'Brien to the chair, and presented the following outline of a case.

About the sixth of last February a young farmer, John D., attempted to jump from a sleigh which stood in front of a butcher's shop, but in doing so, by some accident, he struck against a projecting iron hook such as forms the end of the small iron cranes used by butchers for hanging up quarters of beef and other large pieces of meat. [The instrument was here exhibited.]

The point of the hook caught beneath the upper lid of the right eye, and passing upward usually held the patient suspended in the air, until he could be lifted off and taken down. There was considerable hæmorrhage from the wound and nostrils on that side, and for a few days afterward considerable pain was felt in the head; there was occasional vomiting with some other symptoms such as one would expect to find in a lesion of the brain. These lasted but a short time, however, and when seen by me, Feb. 20, the appearance presented was as follows: Almost complete ptosis of right upper lid, conjunctiva quite œdematous at its upper and inner part, a small granulating tumor marking the point of entrance of the hook.

There was slight divergent strabismus, cornea clear, anterior chamber full, but pupil reacted slowly to light, fundus was obscure and vision slight—only equal to perception of light. There were, however, no brain symptoms; in general patient felt quite well. Three principal points of interest in the case was the fact that the man was neither fatally injured nor was the eye entirely destroyed.

One would naturally suppose that a sharp iron hook of this description would have perforated the roof of the orbit and entering the brain, result in certain death, but although an opening was evidently made into the cranium, yet the result was not serious. This could probably be accounted for by supposing the frontal sinus in this instance to be exceptionally wide. Some discussion followed, after which, reports on prevailing diseases were made by various members. "Malarial fever" appeared to predominate, but the cases were not of special frequence or severity.

Scarlatina was mentioned as being also prevalent, and this was thought by Dr. Rochester to have some relation to the opening of the public schools, which he considered as a fruitful cause of the spread of that disease.

After transacting other routine business the Association adjourned.

